# Peculiar Case of Lithotomy; Singular Nucleus for, &c.

**Published:** 1853-05

**Authors:** 


*Peculiar Case of Lithotomy; singular Nucleus for, and unusual Size of the Calculus.*—The specimen was brought by Dr. Wm. G. Wheeler, of Chelsea, Mass., who performed the operation for its extraction.

Dr. Stedman exhibited the calculus, and gave the following account of the case, furnished by Dr. Wheeler:—

A female, 21 years of age, came under Dr. W.'s care a few months since; she had for a long time suffered severely; all the usual symptoms of stone in the bladder were undeniably present, and, indeed, very marked. A sound being introduced by Dr. W., the presence of a calculus was readily detected. The severity of the local symptoms, and the apparent large size of the stone were the circumstances chiefly engaging attention. The stone seemed immovable, judging by the impression communicated to the sound while examining; the sensation would perhaps convey the idea of the calculus being fixed at certain points. The patient complained occasionally of great pain in the pubic region, as if something sharp pierced the neck of the bladder. Dr. W. was unable to determine the exact size of the calculus. With the hope that it might be sufficiently soft to be crushed, the operation of lithotrity was attempted. With the assistance of Drs. Stedman, of Boston, and Ingalls, of U. S. Marine Hospital, Chelsea, two trials at crushing the stone were made unsuccessfully; the bladder was contracted and irritable, so as to allow of little or no distension by the injection of fluid; and, moreover, the stone proved to be hard, and of such size and shape that it could not be held or fixed within the grasp of the instruments. The constitutional symptoms, also, which followed each of the above attempts, were severe. Dr. W. feared lest inflammation might supervene, of fatal nature, after the last trial.

Disappointed as to lithotrity, the operation of lithotomy seemed the only resource. The case was plainly stated to the friends; a preparatory treatment adopted, and the operation urged. On the 1st of October, the patient, having consented, was etherized, and the operation done by Dr. W., assisted by Drs. Stedman and Ingalls. The vagino-vesical method was chosen, as offering apparently the best chance for the patient, on account of the situation of the stone and the contracted condition of the bladder. The patient being placed in the usual position, an incision was carried downwards and backwards upon the groove of the staff; the operator's forefinger, passed through this opening, felt the stone, of large size, and apparently fixed at certain points. Fears of a sacculated condition of the bladder were suggested; farther exploration by the finger discovered a projecting point, which was at first supposed to be a sharp corner or tubercle of the calculus; but it was finally concluded that it was some foreign body which had served as a nucleus for the calcareous deposit. Efforts to break the stone failed; and, the position of the forceps being changed, one blade was passed over the sharp point alluded to, and after much difficulty, manipulation, and delay, a stone was extracted weighing over two ounces and three-quarters, and there was found passing obliquely *through its centre a large wire hair-pin*, measuring over three inches and a half in length. This pin, and the direction it had with regard to the incision, together with the size of the calculus, caused the delay and embarrassment in the extraction of the latter. Thus is also explained the seeming immobility of the stone at certain points.

The patient aurvives the operation, and has done remarkably well, with the exception that a small fistulous opening still remains. The local as well as the constitutional symptoms have been very mild, when compared with those which followed the previous attempts at lithotrity.

The history of the hair-pin is of some interest, as it gives a probable date of the commencemet of the formation of the calculus. Since the operation, the patient has stated that the pin *was introduced through the urethra aboat six years ago.* She never mentioned this fact to any one; preferring to suffer in silence. The foreign body caused some pain and uneasiness soon after its passage within the bladder, but no severe symptoms were manifested until about two years after its introduction, since which time they have gradually increased in severity

Dr. Charles T. Jackson analysed the stone, and found it to be composed mainly of phosphate of lime, coloured a little with urate of ammonia. The rough surface which the calculus presents is owing to the action of the instrument during the previous efforts to crush it. The points of the hair-pin were bent down upon the side of the stone by the blade of the forceps, thus facilitating the extraction of the mass, and also avoiding laceration of the bladder and adjacent parts, which had suffered so much from continued irritation.

*Four months after the operation.*—Dr. W.'s patient is out, and visits her friends; she has regained her usual health and strength. The fistula still remains, and has resisted all the usual modes of treatment; sutures have not been tried as yet, but will be attepted if required. A few weeks since, the opening seemed likely to close, as little or no water at one time escaped. From cold or some other exciting cause, a small abcess formed within or near the neck of the bladder, which, in evacuating its contents, reopened or enlarged the fistula again.—*American Journal Med. Seience.*

